# The Combined Benefits of Definisse™ Revitalise Threads and Definisse [KP1]^®^ Redensifying Cream Treatments: A 19-Patient Case Series

**DOI:** 10.3390/life16030465

**Published:** 2026-03-12

**Authors:** Adriano Santorelli, Alberto Mentone, Federico Maria D’Alessio, Stefano Uderzo, Martina Manni, Vincent Wong

**Affiliations:** 1 Adriano Santorelli & Partners, Via Raffaele Morghen, 88, 80129 Naples, Italy; 2 Full Face Academy, Via Agostino Depretis, 114, 80133 Naples, Italy; 3 RELIFE Srl, Via Alessandro Marchetti 13 A, 50131 Florence, Italy; 4 Omniere Aesthetics Training Academy, 10 Harley Street, London W1G 9PF, UK

**Keywords:** face rejuvenation, advanced high-precision biostimulation, collagen modulation, peptide-based cosmeceuticals, SA1-III decapeptide

## Abstract

Peptides, particularly SA1-III decapeptide, play a key role in maintaining skin structure by inhibiting collagen degradation. The aim of this analysis was to investigate the combined effects of Definisse™ Revitalise Threads and Definisse [KP1]^®^ Redensifying Cream containing SA1-III for facial rejuvenation. Patients receiving Definisse™ Revitalise Threads were offered Definisse™ Redensifying Cream for one month. Outcomes—including efficacy, safety, and patient satisfaction—were assessed over six months using 3D imaging, clinical evaluation, and the FACE-Q™ questionnaire. Due to the sample size, data were summarised descriptively, with group comparisons using exact Mann–Whitney U tests and paired analyses using the Wilcoxon signed-rank test (*p* < 0.05). Nineteen individuals underwent treatment with Definisse™ Revitalise Threads (17 females and 2 males, aged 38–72 years); 10 patients used additional Definisse [KP1]^®^ Redensifying Cream while 9 did not. All patients reported significant improvements in skin satisfaction, cheek fullness, and perceived aging, based in FACE-Q^TM^ scores before and after treatment. Thread treatment visibly improved facial appearance, but the addition of the peptide-containing cream further enhanced skin quality. Indeed, greater improvements in skin satisfaction were reported by individuals using vs. not using Definisse [KP1]^®^ Redensifying Cream. No adverse events were reported, and the FACE-Q^TM^ Adverse Effects scale indicated only mild, transient effects. In this preliminary series with a six-month follow-up, the combination of Definisse Revitalise Threads and Definisse^®^ Redensifying Cream was well-tolerated and showed potential for enhancing facial rejuvenation. These preliminary findings suggest that peptide-based cosmeceuticals may complement aesthetic procedures and support skin health. These preliminary results will be used to generate hypotheses for larger studies with longer follow-up, which are needed to confirm these effects.

## 1. Introduction

Peptides were first introduced into cosmeceuticals in 1973 with Loren Pickart’s GHK peptide, known for its role in enhancing collagen production and serving as a carrier for Cu(II) [1]. Since then, a wide range of peptides have been developed for treating various skin conditions by influencing collagen turnover. As a crucial structural protein, collagen ensures skin strength and elasticity but naturally declines with age, contributing to wrinkle formation and loss of firmness. Thus, peptides have been used primarily for anti-ageing purposes, addressing oxidative stress, collagen turnover, and fine wrinkles, catering to market demands for youthful skin. These peptides help counteract skin ageing by regulating the balance between protein synthesis and degradation or by temporarily improving skin firmness through neurotransmitter modulation [2].

One notable example is Serpin A1, a neutrophil elastase inhibitor with therapeutic potential for wound healing and skin preservation [3]. This protein primarily targets neutrophil elastase, a protease that degrades components of the extracellular matrix (ECM) to facilitate leukocyte infiltration and inflammation in response to tissue damage. By limiting excessive inflammation, Serpin A1 plays a key role in maintaining wound homeostasis [3].

SA1-III is a bioactive decapeptide derived from the C-terminal portion of Serpin A1 and functions as a signal peptide regulating skin protein turnover. This peptide inhibits protease-driven collagen degradation by blocking neutrophil elastase, a major contributor to ECM breakdown, thereby preserving skin structure and function [2]. SA1-III has been shown to increase collagen levels by reducing its degradation without significantly affecting biosynthesis or cell proliferation, even at low concentrations [2].

Definisse^TM^ [KP1] formulations incorporate SA1-III alongside other bioactive compounds. Both high- and low-molecular-weight hyaluronic acid are ingredients of the cream, serum, and eye contour cream. While both the face serum and face cream contain niacinamide, tocopherol is included in the face cream and eye contour cream. Additionally, the face serum is enriched with active extract of *Tuber magnatum* (white truffle), while the eye contour cream contains cross-linked low-molecular-weight hyaluronic acid.

Definisse^TM^ Revitalise Threads are absorbable monofilament barbed sutures made from poly-L-lactic acid (PLLA) and ε-caprolactone (PCL). PCL provides rubbery properties, while PLLA ensures biocompatibility and biodegradability. Also, PLLA enhances the synthesis of type 1 collagen in a concentration-dependent manner. Clinical studies show that Definisse^TM^ Threads induce subcutaneous fibrosis, lasting beyond 12 months after resorption [4,5], and stimulate fibroblasts, promoting collagen, hyaluronic acid, and elastin synthesis for facial tissue revitalization and repositioning.

At our clinics, we began offering Definisse^TM^ [KP1] Redensifying Cream to patients who were receiving treatment with Definisse^TM^ Revitalise Threads. This article describes the aesthetic outcomes of 19 patients who underwent treatment with Definisse^TM^ Revitalise Threads, some of whom chose to use Definisse^TM^ [KP1] Redensifying Cream.

## 2. Materials and Methods

### 2.1. Subjects

This case series includes individuals from dermatology and aesthetic medicine practices in Italy. Individuals seeking facial rejuvenation with Definisse™ Revitalise Threads (Relife, Menarini Group, Florence, Italy) were treated and their outcomes documented. Before treatment, a clinical evaluation was performed for each subject. Subjects with prior aesthetic treatments, systemic diseases, or medication use were not eligible for the study.

All subjects gave informed consent after an exhaustive explanation of the procedure and its potential adverse events (AEs). Subjects also consented to the use of their photographs in the clinical paper. All costs associated with the clinic appointments and treatments were borne by the clients.

### 2.2. Treatment

All subjects received a treatment with Definisse^TM^ Revitalise Threads. A subgroup of subjects chose to use additional Definisse^TM^ [KP1]^®^ (Relife, Menarini Group, Florence, Italy) Redensifying Cream at home for one month after insertion of the bioabsorbable threads. Briefly, the procedure was performed with the patient in a supine position, targeting the intradermal or superficial subcutaneous layer. After thorough disinfection of the treatment area and anatomical assessment, the target areas were marked to guide thread placement. Barbed threads (5 cm barbed thread—4-0 USP and 60 mm; 23 G cannula needle) were inserted vertically, beginning approximately 1 cm medial to the mandibular angle and advancing towards the temporal region. Each subsequent thread was positioned 1 cm medial to the previous one. To create a supportive mesh network, smooth threads (5 cm smooth thread—6-0 USP and 40 mm; 29 G cannula needle) were introduced horizontally, starting 1 cm superior to the mandibular angle and directed toward the central face. These were spaced 1 cm apart vertically.

**Figure 1:**
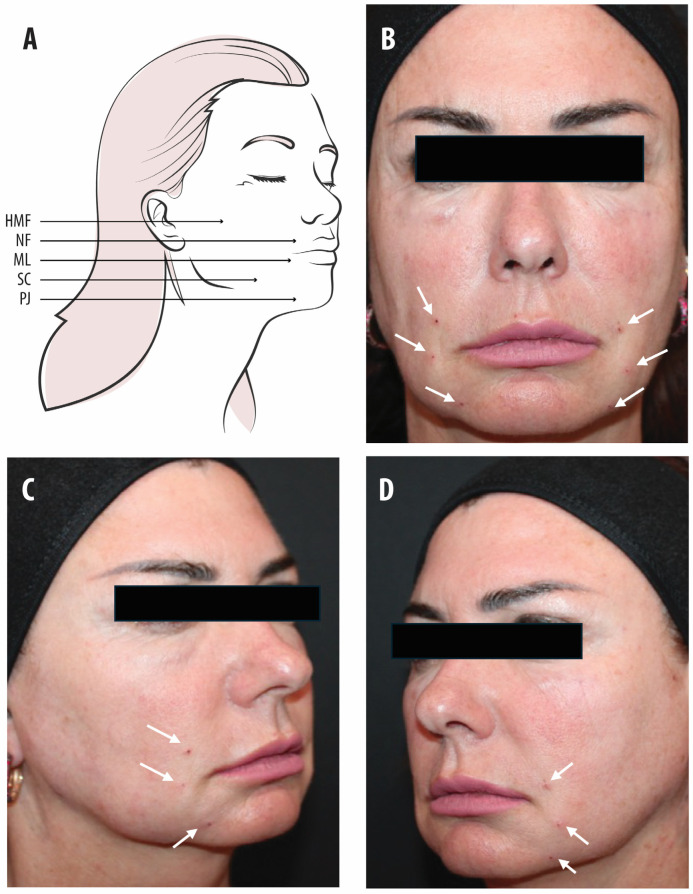
Principal anatomical areas treated. (**A**) Anatomical areas of treatment and (**B–D**) points of intervention. Abbreviations: HMF: hollowed mid face; NF: nasolabial fold; ML: marionette line; SC: sagging cheeks; PJ: pre-jowl sulcus. Arrows indicate insertion areas.

Skin pinching at the insertion site was performed to facilitate accurate placement. Upon completion, ice or cold packs were applied to the treated area for approximately 5 min to minimise swelling and discomfort. Definisse™ Revitalise Thread insertion was performed in the mid-face region (Figure 1) following the application of topical anaesthesia, which was administered at least 30 min prior to the procedure. The individuals who chose to use Definisse^TM^ [KP1] Redensifying Cream were advised to apply it to their face for one month, starting the day after the threads had been inserted. They were advised to apply Definisse^TM^ [KP1] Redensifying Cream to clean the skin in the morning and evening, massaging gently until fully absorbed.

### 2.3. Assessment

Subjects were evaluated before treatment and at a follow-up assessment 6 months post-procedure. Both physicians and subjects independently evaluated the outcomes of the procedure by comparing improvements in facial appearance at the follow-up visit with baseline conditions. Additionally, physicians assessed the occurrence of AEs immediately following the procedure and at the 6-month follow-up visit.

The Vectra H1 imaging system (Canfield Scientific, Parsippany, NJ, USA) was used to capture images before and 1 month after treatment. Utilising compact 3D cameras based on passive stereophotogrammetry, the system captures images from three different angles and seamlessly integrates them into a single 3D representation.

Subjects were asked to complete the FACE-Q^TM^ questionnaire [6] at baseline and at the follow-up visit, 6 months after treatment.

The FACE-Q^TM^ is a validated and reliable patient-reported outcome (PRO) measure designed to assess aesthetic facial procedure outcomes from the patient’s perspective, by evaluating factors important to patients undergoing minimally invasive facial aesthetic treatments [7,8]. The FACE-Q^TM^ follows a modular approach, allowing researchers and clinicians to administer only the most relevant subset of scales based on specific research objectives or patient populations. In this study, subjects were asked to rate: (a) satisfaction with facial appearance for items covering the appearance of the entire face, symmetry and proportion, and how the face looks in photos, bright lights and at the end of the day; (b) ageing appraisal with items about how the respondent feels about the age his/her face looks, including in the mirror and in photos; (c) satisfaction with cheeks with items evaluating the appearance of cheeks (side of the face below cheekbones), including fullness, symmetry and attractiveness. For each independent scale, the scores were calculated as described in the user’s guide [9]. The satisfaction with skin, age appraisal, and satisfaction with cheeks scales each provide independent scores ranging from 0 to 100, with higher scores indicating better outcomes. Individuals who opted to use the cream for one month post-thread insertion were also asked to complete the FACE-Q^TM^ Adverse Effect Face, Cheek, and Neck scale at the 6-month follow-up. This scale ranges from 0 to 100, with higher scores indicating a greater burden of AEs [10]. The smallest detectable change (SDC) in FACE-Q^TM^ score within a group is between 0.95 and 3.23 [8].

### 2.4. Statistical Analysis

Given the small sample size (10 patients using the cream and 9 not using it), all analyses were estimated with non-parametric tests. Comparisons between groups were performed using exact non-parametric Mann–Whitney U tests, while paired analyses were performed used the Wilcoxon signed-rank test. *p*-values < 0.05 were considered statistically significant. All analyses were conducted with SPSS version 30.0 (SPSS, Chicago, IL, USA).

## 3. Results

### 3.1. Case Series

Data were collected on 19 individuals (17 females and 2 males) aged 38 to 72 years who underwent Definisse™ Revitalise Thread treatment at our clinics between 14 and 15 May 2024. Nine individuals were treated with Definisse™ Revitalise Threads only and 10 chose to also use Definisse™ Redensifying Cream. The characteristics of these individuals are shown in Table 1.

At the baseline visit, seborrheic dermatitis was observed in two cases, elastosis in two cases, rosacea in one, and melasma in one (all in the group who opted to use the peptide-containing cream). No other skin conditions or diseases were detected (Table 1).

### 3.2. Outcomes

**Figure 2:**
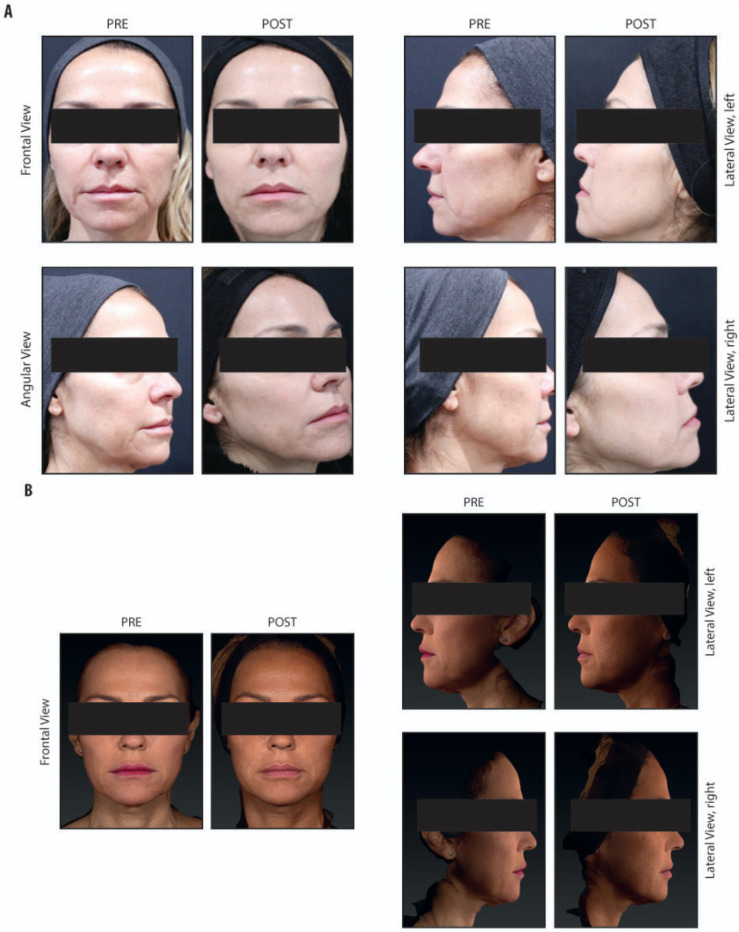
Panels (**A**,**B**) show images of two patients treated with Definisse™ Revitalise Threads in combination with Definisse [KP1]^®^ Redensifying Cream. Images were obtained at rest before the procedure (PRE) and at the 6-month follow-up (POST).

All subjects who underwent Definisse^TM^ Revitalise Thread insertion had noticeable improvements in facial appearance; further improvements in skin quality were seen in the subjects who used Definisse^TM^ [KP1] Redensifying Cream as well (Figure 2).

As shown in Table 2, all subjects—with or without additional peptide-containing cream use—reported improvements at the follow-up visit compared to baseline across all three evaluated FACE-Q^TM^ scales, indicating high levels of satisfaction.

Tangible improvements were seen, with all modules showing an increase in score. The median increase in score before and after treatment was more pronounced in the group that used the peptide-containing cream than in the group that did not (Table 3), particularly for the satisfaction with skin module (*p* = 0.017) and the ageing appraisal module (*p* = 0.023).

### 3.3. Tolerability

No adverse events were reported during or after the treatment, in any of the cases. Additionally, the median FACE-Q^TM^ AE score was 18 (range 24–14) at the 6-month follow-up, suggesting that patients experienced relatively mild AEs (Table 4).

## 4. Discussion

The outcomes among individuals in this case series highlight the synergistic effects of Definisse™ Revitalise Threads and Definisse [KP1]^®^ Redensifying Cream in facial rejuvenation: the addition of [KP1] peptide-based cosmeceuticals significantly enhances skin quality and subject satisfaction.

The FACE-Q^TM^ questionnaire results revealed significant improvements in patient-reported outcomes, particularly in the satisfaction with skin, satisfaction with cheeks, and ageing appraisal scales in both groups. The significantly greater satisfaction with the appearance of the skin among individuals using Definisse [KP1]^®^ Redensifying Cream indicates that the addition of this cosmetic home treatment approach successfully enhanced the improvement of key aesthetic concerns, such as loss of skin elasticity, and the perception of ageing.

The use of SA1-III decapeptide in the Definisse^TM^ [KP1]^®^ formulation appears to be a crucial factor in enhancing treatment efficacy. As a bioactive component, SA1-III plays a pivotal role in inhibiting protease-driven collagen degradation, thereby preserving skin structure and function. This mechanism likely contributes to the prolonged benefits observed in patients receiving the combined treatment. Furthermore, the inclusion of other bioactive compounds such as hyaluronic acid, creatine, niacinamide, and tocopherol enhances hydration, elasticity, and overall skin health. Hyaluronic acid (present in the Definisse [KP1]^®^ formulations) hydrates the superficial layers of the skin, while its low-molecular-weight form penetrates deeper for anti-ageing benefits. Creatine promotes collagen production, minimising wrinkles, while niacinamide (present in the serum and face cream) enhances skin elasticity and smooths fine lines, while white truffle extract (present in the serum) boosts hydration, elasticity, and wrinkle reduction. Acetyl hexapeptide-3 and acetyl tetrapeptide-5 are both present in the eye cream; acetyl hexapeptide-3 diminishes expression lines by inhibiting neurotransmitter release, whereas acetyl tetrapeptide-5 reduces puffiness and dark circles through decongesting action [3]. Use of the Definisse^TM^ [KP1]^®^ formulation containing these ingredients complemented the biostimulation of the Definisse^TM^ Revitalise Threads.

Importantly, our case series supports the safety of the combined treatment. No AEs were reported, and the FACE-Q^TM^ adverse effects scale indicated only mild, transient side effects. This aligns with the existing literature suggesting that both thread-based biostimulation and peptide-based cosmeceuticals are well-tolerated interventions when applied appropriately [2,4,5,11].

It is important to note that this was not a formal study in which subjects were randomised to treatment, and no blinding was used. Therefore, as an observational report, our findings may be affected by noncomparability and bias (e.g., all patients with other skin conditions chose to use the peptide-containing cream). However, the findings are hypothesis-generating and warrant further research in large-scale clinical studies with diverse patient populations, a rigorous design and long-term follow-up to evaluate the effects of this combined approach. Additionally, while the FACE-Q^TM^ questionnaire provides valuable patient-centred insights, objective measures of skin elasticity and collagen density would offer a more comprehensive understanding of the treatment’s biological impact.

## 5. Conclusions

This case series provides preliminary evidence supporting the combined use of Definisse™ Revitalise Threads and Definisse™ Redensifying Cream as a safe and potentially effective strategy for facial rejuvenation. The results suggest that biostimulation can improve facial appearance, while the addition of KP1 peptide-enriched cosmeceuticals may further enhance skin quality, patient satisfaction, and perceived aesthetic outcomes. Improvements in FACE-Q™ scores and the absence of adverse events reinforce the promise of this combination approach in aesthetic medicine.

Given the growing demand for non-invasive, long-lasting rejuvenation strategies, these findings highlight the potential value of integrating cosmeceuticals with procedural interventions to optimise outcomes.

Although limited by a small sample size and non-randomised design—which may introduce selection bias and limit generalizability—this study provides real-world, preliminary insights into safety, efficacy, and patient satisfaction. As an early or pivotal clinical experience, it lays the groundwork for larger, controlled studies with longer follow-up to confirm and extend these observations.

## Figures and Tables

**Table 1:** Characteristics of individuals undergoing thread treatment.

	Thread Treatment Only (n = 9)	Thread Treatment + Peptide-Containing Cream (n = 10)
Sex, n (%)		
Female	7 (77.8)	100 (100.0)
Male	2 (22.2)	0
Age, years		
Median	62	53
Range	45–72	38–71
Facial skin conditions, n (%)		
Seborrheic dermatitis	0	2 (20)
Elastosis	0	2 (20)
Rosacea	0	1 (10)
Melasma	0	1 (10)

**Table 2:** Median FACE-Q satisfaction scores in individuals treated with Definisse™ Revitalise Threads only or in combination with Definisse [KP1]^®^ Redensifying Cream.

	Thread Treatment Only (n = 9)		Thread Treatment + Peptide-Containing Cream (n = 10)	
	Baseline	6 Months	Baseline	6 Months
Satisfaction (skin)				
Median (range)	31 (16–40)	45 (33–55)	25 (9–39)	55 (21–74)
*p*-value	0.008		0.005	
Satisfaction (skin)				
Median (range)	28 (22–47)	59 (43–88)	22.5 (13–44)	63 (55–100)
*p*-value	0.008		0.005	
Satisfaction (skin)				
Median (range)	45 (28–71)	79 (55–95)	42 (0–90)	100 (63–100)
*p*-value	0.008		0.008	

**Table 3:** Difference in median FACE-Q satisfaction scores in individuals treated with Definisse™ Revitalise Threads only or in combination with Definisse [KP1]^®^ Redensifying Cream. Values shown in bold indicate statistical significance.

Score, Median (Range)	Satisfaction Skin			Satisfaction Cheek			Ageing Appraisal		
	Thread Treatment Only (n = 9)	Thread Treatment + Peptide-Containing Cream (n = 10)	*p*-Value	Thread Treatment Only (n = 9)	Thread Treatment + Peptide-Containing Cream (n = 10)	*p*-Value	Thread Treatment Only (n = 9)	Thread Treatment + Peptide-Containing Cream (n = 10)	*p*-Value
Baseline	31 (61–40)	25 (9–39)	0.252	28 (22–47)	22.5 (13–44)	0.287	45 (28–71)	42 (0–90)	0.965
6 months	45 (33–55)	55 (21–74)	**0.017**	59 (22–47)	63 (55–100)	0.248	79 (55–95)	100 (63–100)	**0.023**

**Table 4:** Median scores for adverse events.

	Median	Range	
		Minimum	Maximum
Thread treatment only (n = 9)	16	14	24
Thread treatment + peptide-containing cream (n = 10)	18	15	24
All cases (n = 19)	18	14	24

## Data Availability

Dataset available on request from the authors.

## Abbreviations

3D	three-dimensional
AE	adverse event
ECM	extracellular matrix
PCL	ε-caprolactone
PLLA	poly-L-lactic acid
PRO	patient-reported outcome
SDC	smallest detectable change

## References

[1] Pickart L., Thayer L., Thaler M.M. (1973). A synthetic tripeptide which increases survival of normal liver cells, and stimulates growth in hepatoma cells. Biochem. Biophys. Res. Commun..

[2] Errante F., Ledwoń P., Latajka R., Rovero P., Papini A.M. (2020). Cosmeceutical Peptides in the Framework of Sustainable Wellness Economy. Front. Chem..

[3] Rovero P., Malgapo D.M.H., Sparavigna A., Beilin G., Wong V., Lao M.P. (2022). The Clinical Evidence-Based Paradigm of Topical Anti-Aging Skincare Formulations Enriched with Bio-Active Peptide SA1-III (KP1) as Collagen Modulator: From Bench to Bedside. Clin. Cosmet. Investig. Dermatol..

[4] Santorelli A., Cerullo F., Cirillo P., Cavallini M., Avvedimento S. (2021). Mid-face reshaping using threads with bidirectional convergent barbs: A retrospective study. J. Cosmet. Dermatol..

[5] Wong V. (2021). The Science of Absorbable Poly(L-Lactide-Co-epsilon-Caprolactone) Threads for Soft Tissue Repositioning of the Face: An Evidence-Based Evaluation of Their Physical Properties and Clinical Application. Clin. Cosmet. Investig. Dermatol..

[6] Cogliandro A., Barone M., Persichetti P. (2017). Italian Linguistic Validation of the FACE-Q Instrument. JAMA Facial Plast. Surg..

[7] Klassen A.F., Cano S.J., Scott A., Snell L., Pusic A.L. (2010). Measuring patient-reported outcomes in facial aesthetic patients: Development of the FACE-Q. Facial Plast. Surg..

[8] Gallo L., Rae C., Kim P.J., Voineskos S.H., Thoma A., Pusic A.L., Klassen A.F., Cano S.J. (2024). Establishing test-retest reliability and the smallest detectable change of FACE-Q Aesthetic Module scales. J. Plast. Reconstr. Aesthet. Surg..

[9] Klassen A.F., Cano S.J., Pusic A.L. (2016). FACE-Q Satisfaction with Appearance Scores from Close to 1000 Facial Aesthetic Patients. Plast. Reconstr. Surg..

[10] Klassen A.F., Cano S.J., Schwitzer J.A., Baker S.B., Carruthers A., Carruthers J., Chapas A., Pusic A.L. (2016). Development and Psychometric Validation of the FACE-Q Skin, Lips, and Facial Rhytids Appearance Scales and Adverse Effects Checklists for Cosmetic Procedures. JAMA Dermatol..

[11] Kalyan R., Rafiq N., Wong V. (2017). The Efficacy of Polycaprolactone Threads in Zygomatic and Mandibular Lifting: Consecutive Study from a Single Practitioner’s Experience. Int. J. Clin. Exp. Dermatol..

